# The Diagnostic Value of PI-RADS v2.1 in Patients with a History of Transurethral Resection of the Prostate (TURP)

**DOI:** 10.3390/curroncol29090502

**Published:** 2022-09-05

**Authors:** Jiazhou Liu, Shihang Pan, Liang Dong, Guangyu Wu, Jiayi Wang, Yan Wang, Hongyang Qian, Baijun Dong, Jiahua Pan, Yinjie Zhu, Wei Xue

**Affiliations:** 1 Department of Urology, Ren Ji Hospital, School of Medicine, Shanghai Jiao Tong University, Shanghai 200127, China; 2 Department of Imaging, Ren Ji Hospital, School of Medicine, Shanghai Jiao Tong University, Shanghai 200127, China

**Keywords:** prostate cancer, transurethral resection of the prostate, multiparametric magnetic resonance imaging, prostate-specific antigen

## Abstract

To explore the diagnostic value of the Prostate Imaging–Reporting and Data System version 2.1 (PI-RADS v2.1) for clinically significant prostate cancer (CSPCa) in patients with a history of transurethral resection of the prostate (TURP), we conducted a retrospective study of 102 patients who underwent systematic prostate biopsies with TURP history. ROC analyses and logistic regression analyses were performed to demonstrate the diagnostic value of PI-RADS v2.1 and other clinical characteristics, including PSA and free/total PSA (F/T PSA). Of 102 patients, 43 were diagnosed with CSPCa. In ROC analysis, PSA, F/T PSA, and PI-RADS v2.1 demonstrated significant diagnostic value in detecting CSPCa in our cohort (AUC 0.710 (95%CI 0.608–0.812), AUC 0.768 (95%CI 0.676–0.860), AUC 0.777 (95%CI 0.688–0.867), respectively). Further, PI-RADS v2.1 scores of the peripheral and transitional zones were analyzed separately. In ROC analysis, PI-RADS v2.1 remained valuable in identifying peripheral-zone CSPCa (AUC 0.780 (95%CI 0.665–0.854; *p* < 0.001)) while having limited capability in distinguishing transitional zone lesions (AUC 0.533 (95%CI 0.410–0.557; *p* = 0.594)). PSA and F/T PSA retain significant diagnostic value for CSPCa in patients with TURP history. PI-RADS v2.1 is reliable for detecting peripheral-zone CSPCa but has limited diagnostic value when assessing transitional zone lesions.

## 1. Introduction

Prostate cancer (PCa) is one of the most common malignancies among men worldwide, leading to numerous cancer-related deaths [1,2]. To actively cope with this aggressive global health problem, efforts have been made in the past decades to improve the clinical detection of PCa.

PSA alone as a diagnostic biomarker is insufficient to distinguish PCa from benign prostatic diseases [3]. Studies have shown that at a total PSA level of 4.0 to 10.0 ng/mL, applying the marker of the free/total PSA ratio (F/T PSA) enhances the specificity of PSA testing [4,5]. Advances in imaging techniques have improved the diagnosis of PCa. Multiparametric magnetic resonance imaging (mpMRI) has become an effective noninvasive tool in the assessment of PCa and has demonstrated high value in the detection of clinically significant prostate cancer (CSPCa), defined as Gleason score ≥7 (including 3 + 4 with a prominent but not predominant Gleason 4 component) and/or volume ≥0.5 cc and/or extraprostatic extension (EPE) [6]. To standardize the diagnostic criteria of mpMRI, the Prostate Imaging–Reporting and Data System (PI-RADS) was drafted and lately renewed to version 2.1 [7,8]. A definitive diagnosis of PCa is based on a prostate biopsy. In practice, risk stratification beforehand by serum markers and imaging evaluation has greatly improved cancer detection rates and reduced unnecessary biopsies [9,10,11].

Benign prostatic hyperplasia (BPH) is a progressive disease commonly seen in elderly men and is often addressed by a transurethral resection of the prostate (TURP). We came to notice a certain group of patients in clinical practice who had a surgical history of TURP due to BPH and were suspected of PCa during follow-up visits. On account of the removal of transitional zone tissue during the TURP and possible adenoma regrowth during the follow-up period, serum PSA or F/T PSA testing may be influenced by surgical history. Moreover, with no consensus established yet, whether mpMRI retains diagnostic value also remains uncertain, and patients are, in this case, assigned to prostate biopsies based mainly on clinicians’ judgment, leading to a certain number of unnecessary invasive procedures or delayed diagnoses. Given this situation, we hypothesize that PI-RADS v2.1 may be an effective tool in identifying patients suspected of PCa who require an immediate biopsy.

Therefore, in this present study, we aim to investigate the diagnostic values of PI-RADS v2.1 scores for CSPCa in a cohort of patients with a history of TURP.

## 2. Materials and Methods

### 2.1. Patients

In this retrospective study, consecutive patients who had undergone a 12-core transrectal ultrasound (TRUS)-guided prostate biopsy with previous TURP history at our department between October 2014 and August 2020 were recruited. PSA is reported to drop significantly within 3–6 months after TURP [12,13], while hemorrhage, edema, and early fibrosis at the surgical site after TURP may create biases for mpMRI examinations [14,15]. Therefore, patients who received TURP less than 1 year ago were excluded from the study. The total cohort size was 102. Informed consent was provided by all the participants, and the research was approved by the Institutional Ethical Committee. All patients had a history of receiving a conventional TURP procedure to treat lower urinary tract symptoms due to BPH and had negative pathological results on the removed tissues. Biopsy indication was PSA level > 4 ng/mL or suspected digital rectal exam results. Exclusion criteria consisted of patients with previous local treatment of the prostate other than TURP and patients with positive pathological reports prior to our procedure. Each patient drew blood for serum PSA and F/T PSA and underwent an mpMRI examination before the biopsy.

### 2.2. MRI and Reporting Protocol

All mpMRI included T2W, DW, and DCE imaging sequences. Two experienced genitourinary radiologists blinded to the clinical details reviewed and reported readouts following the standards of PI-RADS v2.1 [7,8]. Since the assessment of the transitional zone (TZ) and peripheral zone (PZ) relies on different key sequences of imaging, PI-RADS scores were proposed for each prostate zone separately for each patient. A single PI-RADS score of a patient represents the PI-RADS score of the dominant lesion in the whole gland, while PI-RADS TZ and PI-RADS PZ scores represent the PI-RADS score of the dominant lesion in the TZ and PZ, respectively.

### 2.3. Biopsy Protocol

All patients underwent a 12-core transrectal ultrasound (TRUS)-guided transperineal prostate biopsy. The well-designed biopsy template covers the bilateral anterior TZ, posterior TZ, anterior horn of PZ, anterior lateral PZ, posterior lateral PZ, and posterior medial PZ. The biopsy template is shown in Figure S1. Cores No. 1–8 are targeted to the PZ of the prostate, while Cores No. 9–12 are targeted to the TZ. Slight adjustments were made to adapt to the tissue defect caused by TURP and to cover all suspected lesions found in the imaging. All biopsies were performed by a single experienced urologist, and all samples were reviewed by a single specialized uropathologist to conclude the definitive diagnosis. Clinically significant cancer was defined following the PI-RADS V2.1 guidelines [7,8] as PCa with a histologic Gleason score ≥7 (including 3 + 4 with a prominent but not predominant Gleason 4 component) and/or volume ≥0.5 cc and/or extraprostatic extension (EPE).

### 2.4. Statistical Analysis

Statistical analyses were performed using SPSS version 26.0. Statistical significance was set as *p* < 0.05. We used the Mann–Whitney rank sum test for nonparametric variables and Fisher’s exact chi-square test for categorical variables. Receiver operating characteristic (ROC) analyses were performed using biopsy results as the gold standard to reflect the diagnostic performance of PSA, F/T PSA, and PI-RADS v2.1. ROC curves were compared using the DeLong test. The area under the curve (AUC) and Youden’s index were calculated. Logistic regression analyses were conducted to explore the predictive values of the variables, and the odds ratios (ORs) were computed to quantify the predictive ability of the factors.

## 3. Results

A total of 102 patients were included in the study. The median age was 73.5 years (interquartile range; IQR 68–78 years), the median time after TURP was 8 years (IQR 4–10.25 years), the median PSA level was 12.3 ng/mL (IQR 7.41–19.26 ng/mL), and the median F/T PSA was 0.15 (IQR 0.11–0.20). In all, 56 patients were diagnosed with PCa, among which 43 patients had CSPCa, while a Gleason score of 3 + 3 = 6 was present in the remaining 13. Table 1 shows the characteristics of patients with CSPCa and non-CSPCa or negative biopsy. While no significant difference was found in time after TURP between the two groups, patients presenting with CSPCa exhibited significantly older age (*p* = 0.000), higher PSA levels (*p* = 0.006), and lower F/T PSA (*p* = 0.000).

**Figure 1:**
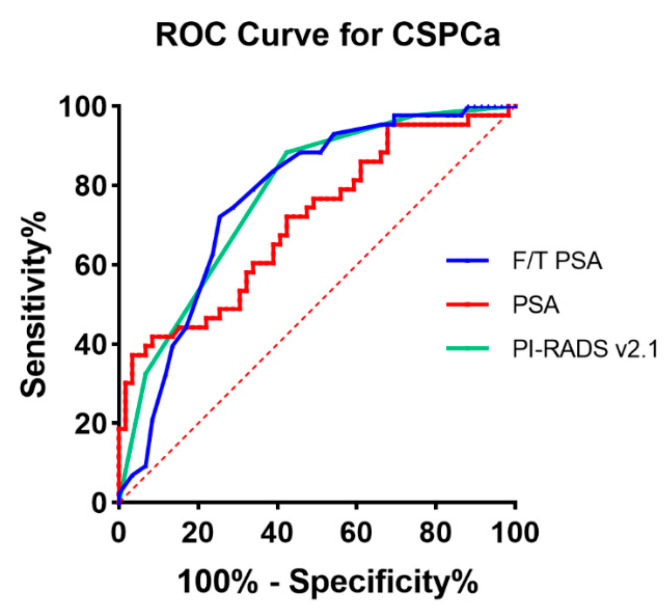
ROC curves of PSA, F/T PSA, and PI-RADS v2.1 in predicting CSPCa.

ROC curves were constructed to determine the diagnostic value of PSA, F/T PSA, and PI-RADS v2.1 in our cohort (Figure 1). The area-under-the-curve (AUC) value of PSA, F/T PSA, and PI-RADS v2.1 for predicting CSPCa was 0.710 (95 CI% 0.608–0.812), 0.768 (95 CI% 0.676–0.860), and 0.777 (95 CI% 0.688–0.867), respectively. A comparison of the three ROC curves showed no statistically significant differences. Setting the threshold at 23.81 ng/mL, PSA showed the best Youden’s index score of 0.338, with the sensitivity, specificity, PPV, and NPV of 37.2%, 96.6%, 88.9%, and 67.9%, respectively, in differentiating CSPCa from this cohort. At a cutoff value of 10 ng/mL, PSA showed a sensitivity value of 76.7% (but a specificity of only 45.8%) and a PPV and NPV of 76.7% and 73.7% in differentiating CSPCa from this cohort. The best cutoff value for F/T PSA was obtained at 0.135. At this level, Youden’s index, sensitivity, specificity, PPV, and NPV for F/T PSA were 0.467, 72.1%, 74.6%, 67.4%, and 78.6%. For PI-RADS v2.1, when the cutoff value is set as ≥4, the best Youden’s index, sensitivity, specificity, PPV, and NPV were 0.460, 88.4%, 57.6%, 60.3%, and 87.2%, respectively. If the cutoff value is set as ≥3, the sensitivity, specificity, PPV, and NPV were 97.7%, 25.4%, 48.8%, and 93.8%, respectively.

In univariate logistic regression analysis, age, PSA, F/T PSA (<0.135 vs. ≥0.135), and PI-RADS v2.1(≥3 vs. <3) showed significant associations with the biopsy results. However, in multivariate logistic regression analysis, only age, F/T PSA, and PI-RADS v2.1 remained independent predictors for CSPCa (Table S1).

**Figure 2:**
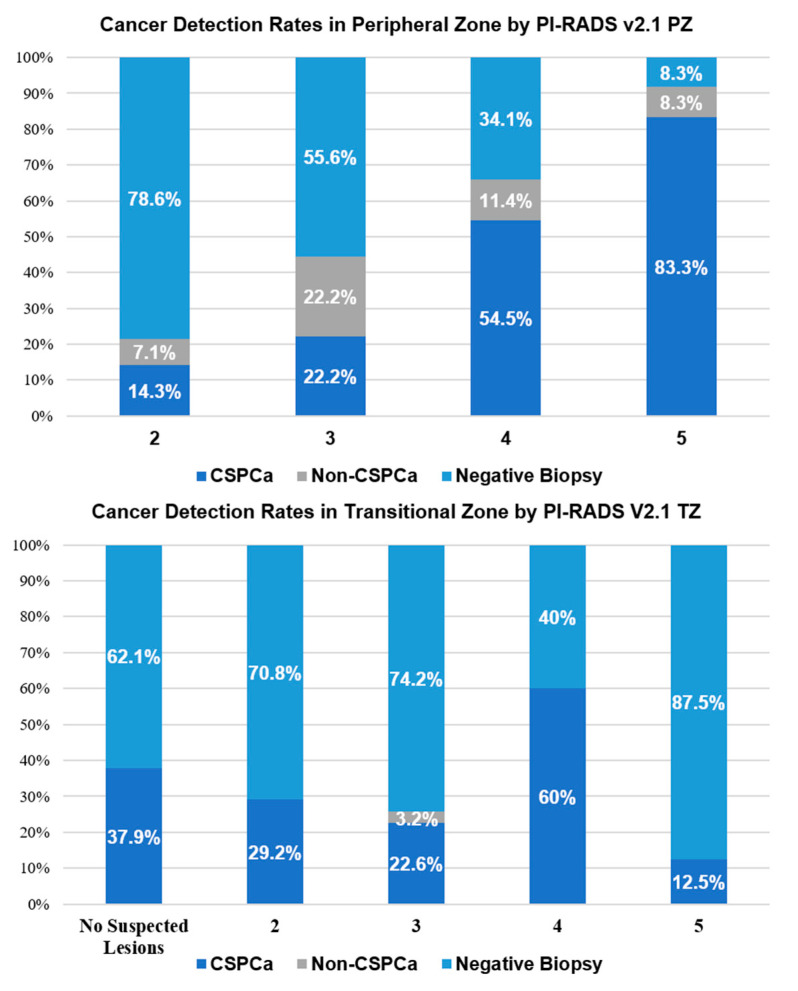
CSPCa detection rates by PI-RADS v2.1 scores.

Among the 43 patients with CSPCa biopsy results, 31 had CSPCa detected from both PZ and TZ cores, 11 had CSPCa detected only in the PZ, and 1 had CSPCa detected only in the TZ. The PI-RADS v2.1 TZ score and the PI-RADS v2.1 PZ score were proposed and analyzed (Table 2). There was a significant difference in PI-RADS v2.1 scores between patients with or without peripheral-zone CSPCa. Such a difference was not observed in the TZ subgroup, indicating PI-RADS v2.1 may not be an effective tool to diagnose CSPCa in the TZ in this cohort. Figure 2 shows the biopsy results by PI-RADS v2.1 TZ and PZ scores. For PI-RADS PZ scores of 2, 3, 4, and 5 in the peripheral zone, the CSPCa detection rates were 14.3%, 22.2%, 54.5%, and 83.3%, respectively.

**Figure 3:**
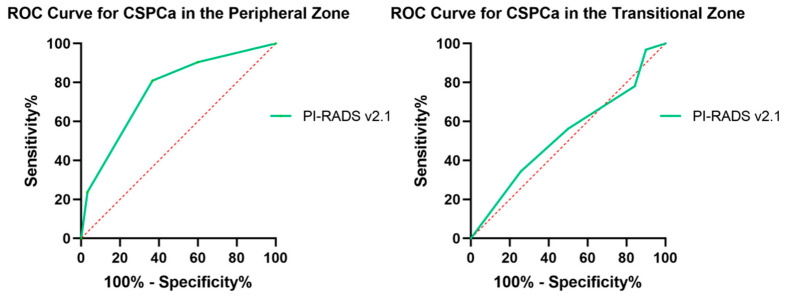
ROC curves of PI-RADS v2.1 in predicting CSPCa in the peripheral zone and the transitional zone.

In ROC analysis (Figure 3), PI-RADS v2.1 PZ obtained significant diagnostic value in the peripheral zone (AUC 0.780 (95%CI 0.665–0.854; *p* < 0.001)). At a cutoff value of ≥3, PI-RADS v2.1 PZ had the sensitivity, specificity, PPV, and NPV of 90.5%, 40.0%, 51.3%, and 85.7%, respectively. However, in the transitional zone, PI-RADS v2.1 TZ demonstrated no diagnostic value for CSPCa (AUC 0.533 (95%CI 0.410–0.557; *p* = 0.594)).

In univariate logistic regression analysis (Table S2), compared to PI-RADS PZ score 2, the odds ratio was 7.200 (95%CI 2.140–24.230) for patients with a PI-RADS PZ score of 4 and 30.000 (95%CI 4.714–190.939) for patients with a PI-RADS PZ score of 5. PI-RADS PZ score ≥3 was an independent predictor for CSPCa (OR = 6.333, 95%CI 2.000–20.052, *p* = 0.002). As for the transitional zone, PI-RADS TZ scores of 2, 3, 4, and 5 showed no significant odds ratio compared to no suspected lesions by PI-RADS v2.1. PI-RADS TZ score ≥3 showed limited significance for predicting CSPCa (OR = 0.778, 95%CI 0.335–1.803, *p* = 0.558).

**Figure 4:**
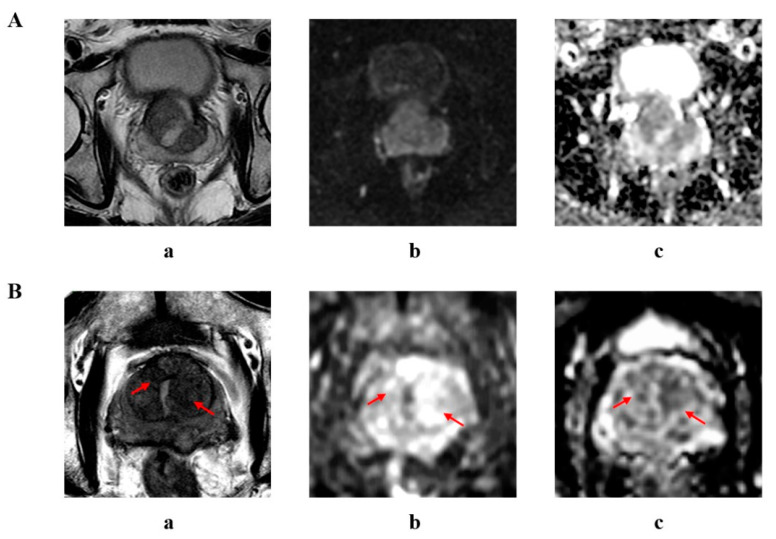
mpMRI results of two biopsy patients with TURP history. (**A**) A 63-year-old patient previously received TURP; no lesions with PI-RADS score ≥ 3 were found in TZ. Biopsy results showed CSPCa with a Gleason score of 4 + 3 = 7 in the TZ. (**B**). A 74-year-old patient previously received TURP; two lesions classified as PI-RADS score 4 were found in the bilateral TZ (arrow). Biopsy results were negative in the TZ. (a) T2-weighted image; (b) DWI with b-value of 1500 s/mm^2^; (c) ADC map.

Figure 4 shows the mpMRI results of two patients. For both patients, the PI-RADS v2.1 scoring system demonstrated unsatisfactory diagnostic value in the transitional zone.

## 4. Discussion

Benign prostatic hyperplasia (BPH) is a condition with high prevalence among aged men; 50% of men with BPH develop LUTS that require medical intervention [16]. Transurethral resection of the prostate (TURP) has remained the cornerstone of BPH surgical treatment for decades. The TURP operation removes tissue from the TZ of the gland to address BPH-related obstruction, during which the PZ of the gland is not resected. It is reported that a secondary TURP is required to address the re-developed prostatic obstruction for 2.9%, 5.8%, and 7.4% of patients in 1, 5, and 8 years after primary TURP [17], which indicates a significant proportion of patients may experience adenoma regrowth in the resected TZ of the prostate after a TURP procedure. Therefore, patients are still at risk of PCa in both the PZ and the TZ of the gland after receiving a TURP. Although patients with a history of TURP are commonly seen in a urology clinic, few studies have addressed the clinical characteristics of this cohort. Clinical studies regarding diagnostic tests for PCa commonly set patients with prostatic surgery history into the exclusion criteria, leading to the scarcity of evidence for the diagnostic value of PSA, F/T PSA, or mpMRI results in this cohort. We noticed the lack of references for patients suspected of PCa who also have a history of TURP, leading to our study being the first to look into this issue.

PSA levels can be affected by prostate volume or the presence of BPH or prostatitis. Aus et al. [13] reported that in 190 patients who underwent TURP due to BPH, the mean PSA levels were reduced by 70%, from 6.0 to 1.9 ng/mL 3–4 months after TURP, while the mean prostate volume was reduced by 58%, from 63.3 to 26.5 cc. Furuya et al. [18] found that the removal of 1 g of BPH tissue reduced serum PSA levels by an average of 0.18 ng/mL, revealing the correlation between serum PSA levels and the TZ volume. A TURP procedure removes BPH tissue from the TZ, resulting in a significant decrease in PSA, while the possible regrowth of adenoma at the surgery site may lead to an increase in PSA levels in the long term after surgery. Taken together, the baseline PSA levels appear uncertain in this cohort. Our study revealed positive results in the diagnostic value of the absolute PSA level in CSPCa in this cohort. Setting the cutoff value at 10 ng/mL, PSA shows high sensitivity (76.7%) but poor specificity (45.8%). 

Catalona et al. [5] reported that at a 25% cutoff, F/T PSA detected 95% of cancers while avoiding 20% of unnecessary biopsies for patients with a PSA level of 4.0 to 10.0 ng/mL. The reasons for the changes in F/T PSA in PCa are not fully understood [19]. Recker et al. [20] explored the changes in serum total and free PSA in TURP patients and revealed that despite a decline in t-PSA by 72% post-TURP, F/T PSA remained stable (median 24.9% pre-op vs. 26.6% post-op), indicating the potential of F/T PSA in PCa detection after TURP. Consistent with their results, our study revealed the high diagnostic value of F/T PSA in CSPCa. At a cutoff value of 13.5%, the sensitivity and specificity in the cohort were 72.1% and 74.6%, respectively, and an F/T PSA under 13.5% indicated a nearly 9-fold higher risk for CSPCa.

PI-RADS v2.1 is a structured reporting system for standardizing the mpMRI results for detecting PCa. Our study revealed that PI-RADS v2.1 is a reliable tool for CSPCa detection in patients with a history of TURP. A cutoff at PI-RADS score ≥4 resulted in 88.4% of CSPCa cases found and 57.6% of unnecessary biopsies avoided. To further explore the value of PI-RADS V2.1, we reviewed the imaging scores of two prostatic zones separately. The results showed that PI-RADS V2.1 remains reliable in identifying CSPCa in the PZ. Rudolph et al. [21] reported CSPCa detection rates of 13.0%, 10.0%, 42.9%, and 68.3% for PI-RADS v2.1 scores of 2, 3, 4, and 5, respectively, in the PZ. The detection rates in the PZ of our patients are consistent with earlier studies of PI-RADS in the whole patient population [22,23,24,25,26], indicating that the PZ CSPCa lesions were not affected by the excision or regrowth of prostatic tissue. Therefore, when managing patients with a history of TURP, mpMRI can provide important references for PZ lesions, and a high PI-RADS PZ score should be an indication for biopsy.

Concerning the TZ, the performance of CSPCa detection was limited with PI-RADS v2.1 scores. For lesions with a PI-RADS v2.1 score of 2, 3, 4, and 5, CSPCa detection rates were reported to be 7.4%, 8.3%, 40.0%, and 61.7%, respectively, in the general biopsy patient population [21], which differ significantly from our findings in patients with TURP history. This result might be due to the tissue composition changes secondary to TURP, such as inflammatory tissue reactions consisting of mononuclear cells and giant cells [27] or a mixture of scar tissue, glandular tissue, and stromal tissue, which are commonly presented as areas with hypointensity in T2WI. In this instance, corresponding changes in T2W signals can easily mimic the diagnostic criteria for PI-RADS 4, which creates challenges for accurate imaging assessment [8]. From our results, mpMRI should not be referenced when assessing transitional zone lesions, and PI-RADS TZ scores should not be decisive factors when considering a biopsy.

To our knowledge, our study is the first study to probe into the diagnostic tests for CSPCa in patients with TURP history. This study has some limitations. First, it is a retrospective, single-center study with a relatively small sample size. Clinical characteristics of our cohort show great heterogeneity, especially in the time after TURP and PSA levels, which may reduce the representativeness of our study cohort. The small sample size limited the reliability of subgroup analysis. Studies [28,29] have suggested the monitoring of PSA dynamic changes or PSA velocity as a predictor for PCa in post-TURP patients. Pre-TURP PSA levels may provide reference cutoff values for our study. Unfortunately, due to the long interval since TURP and the lack of follow-up data in our patient cohort, such information was not available. PSA-density, as well as MRI-estimated lesion volume, has been reported to provide additional predictive value to the detection of CSPCa in the whole patient population [30,31,32]. However, impaired anatomy at the previous TURP site created difficulties for prostate and lesion volume measurements. The clinical implications of our findings remain inconclusive. Further large-sample prospective studies are needed to conclude the characteristics of such a cohort and explore optimal diagnostic procedures and treatment strategies.

## 5. Conclusions

Our study reveals that PSA and F/T PSA retain significant diagnostic value for CSPCa in patients with TURP history. PI-RADS v2.1 is reliable for detecting CSPCa in the PZ but has limited diagnostic value when assessing TZ lesions.

## Figures and Tables

**Table 1:** Patient characteristics in different biopsy results.

	CSPCa	Non-CSPCa or Negative Biopsy	*p*-Value
Age (yrs), median (IQR)	77 (72–80)	70 (66–75)	0.000
Time after TURP (yrs), median (IQR)	6 (3–12)	8 (5–10)	0.557
PSA (ng/mL), median (IQR)	14.73 (10.97–36.00)	10.91 (6.19–15.89)	0.000
F/T PSA, median (IQR)	0.12 (0.09–0.15)	0.18 (0.13–0.23)	0.000
PI-RADS V2.1 n (%)			
2	1 (2.3)	15 (25.4)	0.000
3	4 (9.3)	19 (32.2)	
4	24 (55.8)	21 (35.6)	
5	14 (32.6)	4 (6.8)	

**Table 2:** PI-RADS v2.1 scores for the peripheral zone and the transitional zone.

	CSPCa	Non-CSPCa/Negative Biopsy	*p*-Value
**PI-RADS v2.1 PZ n (%)**			
2	4 (9.5%)	24 (40.0%)	0.000
3	4 (9.5%)	14 (23.3%)	
4	24 (57.1%)	20 (33.3%)	
5	10 (23.8%)	2 (3.3%)	
**PI-RADS v2.1 TZ n (%)**			
**No Suspected Lesions**	11 (34.4%)	18 (25.7%)	0.167
2	7 (21.9%)	17 (24.3%)	
3	7 (21.9%)	24 (34.3%)	
4	6 (18.8%)	4 (5.7%)	
5	1 (3.1%)	7 (10.0%)	

## Data Availability

The data presented in this study are available upon request from the corresponding author. The data are not publicly available due to the privacy of patients.

## Supplementary Materials

The following supporting information can be downloaded at: https://www.mdpi.com/article/10.3390/curroncol29090502/s1, Figure S1: 12-core transrectal ultrasound (TRUS)-guided transperineal prostate biopsy template; Table S1: Univariate and multivariate logistic regression analysis for CSPCa; Table S2: Logistic regression analysis of PI-RADS v2.1 in the peripheral and transitional zones.
