# Supplementary material for: The Diagnostic Value of PI-RADS v2.1 in Patients with a History of Transurethral Resection of the Prostate (TURP)
Source: Curr Oncol. 2022 Sep 5;29(9):6373–82. doi: 10.3390/curroncol29090502 (PMC9497547; doi:10.3390/curroncol29090502)
Supplement: Supplementary file 1 [file curroncol-29-00502-s001.zip › curroncol-1862900-supplementary.pdf]

Current Oncology

MDPI

Supplementary Material

The Diagnostic Value of PI-RADS v2.1 in Patients with a History of Transurethral Resection of the Prostate (TURP)

Jiazhou Liu [1,†], Shihang Pan [2,†], Liang Dong [1,†], Guangyu Wu [2], Jiayi Wang [1], Yan Wang [1], Hongyang Qian [1], Baijun Dong [1], Jiahua Pan [1], Yinjie Zhu [1,*] and Wei Xue [1,*]

[1] Department of Urology, Ren Ji Hospital, Shanghai Jiao Tong University School of Medicine, Shanghai 200127, China
[2] Department of Imaging, Ren Ji Hospital, Shanghai Jiao Tong University School of Medicine, Shanghai 200127, China
* Correspondence: zhuyinjie@renji.com (Y.Z.); xuewei@renji.com (W.X.); Tel.: +86-21-68383757 (Y.Z. & W.X.); Fax: +86-21-58394262 (Y.Z. & W.X.)
† These authors equally contribute to the study.

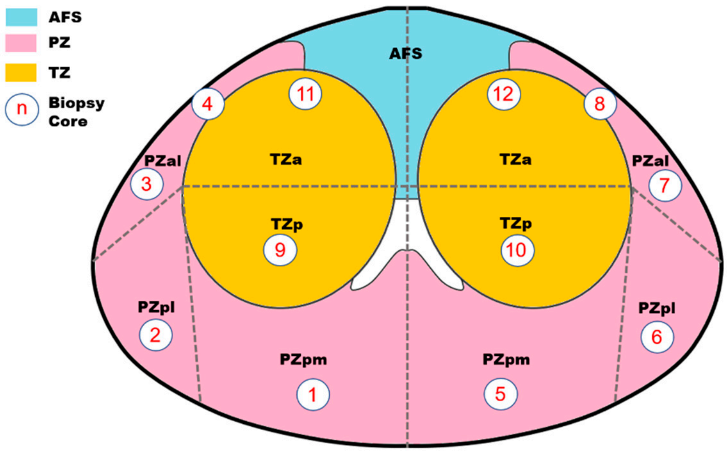

Figure S1. 12-core transrectal ultrasound (TRUS)-guided transperineal prostate biopsy template. Transverse view of prostate gland. 12 biopsy cores distribute bilaterally to cover the peripheral zone and transitional zone. AFS: anterior fibromuscular stroma; TZa: anterior transitional zone; TZp: posterior transitional zone; PZal: anterior lateral peripheral zone; PZpl: posterior lateral peripheral zone; PZpm: posterior medial peripheral zone.

Table S1. Univariate and multivariate logistic regression analysis for CSPCa.

**Variables**	**Categories**	**Univariate Analysis**		**Multivariate Analysis**	
		**OR (95%CI)**	***p* Value**	**OR (95%CI)**	***p* Value**
Age	continuous	1.144 (1.061–1.234)	0.000	1.116 (1.017–1.225)	0.020
PSA	continuous	1.070 (1.025–1.118)	0.002	1.043 (0.998–1.091)	0.061
F/T PSA	<0.135 vs. ≥0.135	7.578 (3.120–18.405)	0.000	8.158 (2.783–23.912)	0.000
PI-RADS V2.1	≥3 vs. <3	14.318 (1.810–113.234)	0.000	13.070 (1.462–116.823)	0.021

Table S2. Logistic regression analysis of PI-RADS v2.1 in the peripheral zone and transitional zone.

	**Peripheral Zone**	
**PI-RADS v2.1 PZ**	**OR (95%CI)**	***p* Value**
2	1 (Reference)	
3	1.714 (0.369–7.954)	0.491
4	7.200 (2.140–24.230)	0.001
5	30.000 (4.714–190.939)	0.000
**≥3 vs. <3**	6.333 (2.000–20.052)	0.002
	**Transitional Zone**	
**PI-RADS v2.1 TZ**	OR (95%CI)	***p* Value**
**No Suspected Lesions**	1 (Reference)	
2	0.674 (0.212–2.142)	0.503
3	0.477 (0.155–1.474)	0.199
4	2.455 (0.564–10.684)	0.231
5	0.234 (0.025–2.164)	0.201
**≥3 vs. <3**	0.778 (0.335–1.803)	0.558
